# Nerve Growth Factor in Cancer Cell Death and Survival

**DOI:** 10.3390/cancers3010510

**Published:** 2011-02-01

**Authors:** Niamh H. Molloy, Danielle E. Read, Adrienne M. Gorman

**Affiliations:** Apoptosis Research Centre, School of Natural Sciences, National University of Ireland, Galway, Ireland; E-Mails: n.molloy1@nuigalway.ie (N.H.M.); d.read@nuigalway.ie (D.E.R.)

**Keywords:** apoptosis, breast cancer, nerve growth factor (NGF), p75 neurotrophin receptor (p75^NTR^), prostate cancer, TrkA

## Abstract

One of the major challenges for cancer therapeutics is the resistance of many tumor cells to induction of cell death due to pro-survival signaling in the cancer cells. Here we review the growing literature which shows that neurotrophins contribute to pro-survival signaling in many different types of cancer. In particular, nerve growth factor, the archetypal neurotrophin, has been shown to play a role in tumorigenesis over the past decade. Nerve growth factor mediates its effects through its two cognate receptors, TrkA, a receptor tyrosine kinase and p75^NTR^, a member of the death receptor superfamily. Depending on the tumor origin, pro-survival signaling can be mediated by TrkA receptors or by p75^NTR^. For example, in breast cancer the aberrant expression of nerve growth factor stimulates proliferative signaling through TrkA and pro-survival signaling through p75^NTR^. This latter signaling through p75^NTR^ promotes increased resistance to the induction of cell death by chemotherapeutic treatments. In contrast, in prostate cells the p75^NTR^ mediates cell death and prevents metastasis. In prostate cancer, expression of this receptor is lost, which contributes to tumor progression by allowing cells to survive, proliferate and metastasize. This review focuses on our current knowledge of neurotrophin signaling in cancer, with a particular emphasis on nerve growth factor regulation of cell death and survival in cancer.

## 1. Introduction

Understanding the processes that regulate cell death and survival is essential for the development of therapeutic strategies for the treatment of cancer. In this regard, targeting growth factors has particular significance. Here we discuss evidence regarding the role of nerve growth factor (NGF), the first growth factor discovered, in cancer biology with particular focus on its role in the death and survival of cancer cells.

The balance between cell death and survival in an organism is key to maintaining tissue homeostasis. Cancers can arise as a result of many changes in the behavior of cells, one of which is their prolonged survival beyond normal limits, or their acquired ability to evade cell death in the face of stresses that would lead to the death and removal of a normal cell [1]. Some of the cell death pathways that are affected include apoptotic responses and autophagy. Apoptosis is a term coined in 1972 to describe a naturally occurring programmed cell death mechanism [2]. As well as its induction by cytotoxic stimuli, apoptosis normally helps to maintain a constant cell number in multicellular organisms. Some of the morphological characteristics of apoptotic cell death include cell shrinkage, chromatin condensation, nuclear fragmentation, membrane blebbing, formation of apoptotic bodies and finally, disposal of cellular debris by phagocytes. These steps are highly controlled and executed in an ordered fashion [3]. The morphological characteristics of apoptosis are largely mediated by caspases, a family of *c*ysteine-dependent *aspartic* acid specific prote*ases*, which cleave many different proteins to bring about controlled cell death [4,5]. These are activated in a cascade, with initiator caspases activating downstream effector caspases through proteolysis [4]. Autophagy can act as both a pro-survival and pro-death process in multicellular organisms. It is a normal response to nutrient deprivation and certain other stresses whereby cells initiate auto-digestion pathways to release alternative sources of energy and promote cell survival. However, excessive autophagy is linked to cell death and is sometimes called autophagic cell death [6,7]. Autophagic cell death can be utilized when apoptotic pathways are inaccessible [8-10]. Autophagic cell death is caspase-independent [11,12]. It involves engulfment of cellular components and organelles into double-membraned vesicles called autophagosomes which fuse with lysosomes to produce autophagolysosomes, where the contents are subsequently degraded [7,13].

Many growth factors and cytokines are important for cell death and survival signaling, and dysregulation of growth factor signaling often plays an important role in cancer development [1]. Moreover, progression of cancer, including development of metastasis and angiogenesis of tumors, is often found to be linked to altered expression of growth factors and their receptors, many of which are known oncogenes and tumor suppressors [14]. NGF is one such protein and during recent years, NGF signaling has been shown to alter cell death and survival in various cancer cells. Here we will describe the role of NGF in cell death and survival signaling with a particular focus on breast and prostate cancers, since most advances in this area have been made in these cancers.

## 2. Neurotrophins and Nerve Growth Factor (NGF)

The discovery by Rita Levi-Montalcini in the 1940s of the first growth factor pioneered the field of growth factor research. She identified NGF as a substance secreted from mouse sarcoma tissue that stimulated neuronal survival and neurite outgrowth from chicken ganglia. This provided some of the first evidence of paracrine signaling, whereby cells in one tissue secreted a protein which readily diffused to another tissue to elicit cellular changes in the target tissue [15]. NGF is the prototypic member of the small family of neurotrophins, which also includes brain-derived neurotrophic factor (BDNF), neurotrophin-3 (NT-3) and NT-4/5 [16,17]. The history of NGF and neurotrophin research is tightly woven into the field of neuroscience, where NGF has been shown to promote survival and differentiation of neurons, outgrowth of neurites, while BDNF is also involved in learning and memory [18-20]. A critical role for neurotrophins in development of the nervous system has been revealed by gene targeting in the mouse [21-23]. However, it is pertinent here to remember that NGF was first shown to be secreted from mouse sarcoma tissue and that neurotrophins are expressed in many areas of the body and frequently participate in signaling beyond the nervous system [18]. In fact, the non-neural functions of NGF have gained significant attention in recent years and NGF has been shown to have roles in development of the male and female reproductive systems, the endocrine, cardiovascular and immune systems [21]. It is recognized that dysregulation of neurotrophin signaling plays important roles in the pathogenesis of many tumors including several of non-neural origin [14,24]. In these cancers, NGF and other neurotrophins regulate cell proliferation and invasion as well as cell death and survival. These various responses are dependent on cell type and often reflect the expression of receptors and adaptor proteins by the cells.

### 2.1. Neurotrophin Receptors

**Figure 1.**
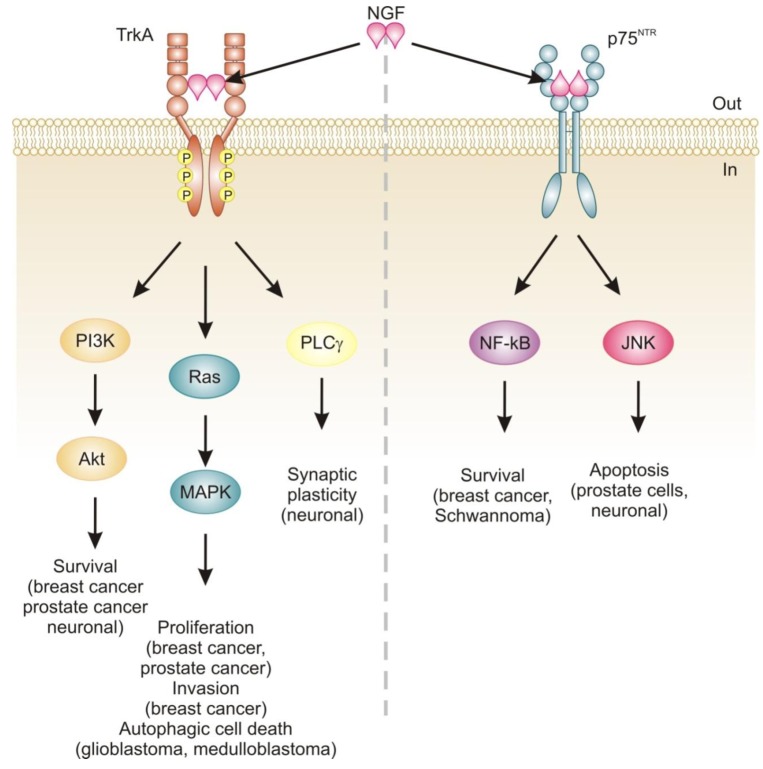
Signaling pathways activated by nerve growth factor (NGF). NGF binding to TrkA receptor mediates proliferation, differentiation and survival via activation of PI3K/Akt, Ras/MAPK and PLCγ pathways. This extends to driving invasion, metastasis and autophagic cell death in certain cancer cell types. NGF binding to the p75^NTR^ receptor initiates recruitment of various adaptors, which activate NF-κB and c-Jun N-terminal kinase (JNK). These mediate opposing effects of survival and apoptosis respectively. PI3K, Phosphatidylinositol-3-kinase; MAPK, Mitogen activated protein kinase; PLCγ, Phospholipase Cγ; NF-κB, Nuclear factor-κB; JNK, c-Jun N-terminal kinase.

Neurotrophins mediate their diverse functions through two structurally distinct classes of transmembrane receptors; the ‘common’ p75 neurotrophin receptor (p75^NTR^) which binds all of the neurotrophins with approximately equal affinity, and specific receptor tyrosine kinase receptors called tropomyosin related kinases (Trks) that exhibit specificity in neurotrophin binding (Figure 1) [18,25].

p75^NTR^ is a member of the death-promoting tumor necrosis factor receptor (TNF-R) superfamily, which also includes the characteristic death receptors TNF-R apoptosis-inducing ligand (TRAIL)-R and Fas/CD95 [26,27]. p75^NTR^ is a 75 kDa glycoprotein with four extracellular cysteine rich repeats required for ligand binding [28,29]. It is a single pass type I transmembrane receptor, with an intracellular domain containing a juxtamembrane region and a type II consensus death domain (DD) sequence [18]. Although p75 binds dimeric neurotrophin ligands, there is some controversy over the oligomeric status of p75 and evidence indicates that it may signal as a monomer or as a dimer [30]. Recently, it was shown that p75 can form covalent homodimers through a disulphide bond in the transmembrane region. In this case, neurotrophin binding is understood to induce a conformational change in the receptor, such that it pivots on this disulfide bond at Cys257 to permit access of intracellular adaptor proteins to the intracellular domain in what is termed a snail-tong model [30-32]. The receptor does not possess intrinsic enzymatic activity and transduces signals through recruitment of a variety of adaptor proteins to the intracellular domain, leading to proliferation, survival, or cell death [18,33]. p75^NTR^-mediated downstream signal transduction pathways are extremely diverse, being heavily dependent on the cell context, availability of intracellular adaptor molecules and expression of interacting transmembrane co-receptors [34]. Interestingly, p75^NTR^ also serves as a receptor for immature pro-neurotrophins which induce cell death in a manner that is dependent on binding to a co-receptor, sortilin [35]. Furthermore, p75^NTR^ is capable of eliciting cellular signals in the absence of bound ligand [31]. p75^NTR^ signaling can also lead to downstream activation of nuclear factor-kappa B (NF-κB) which promotes cell survival through upregulation of anti-apoptotic genes such as cFLIP, which interferes with the activation of initiator caspase-8, the Bcl-2 family member Bcl-X_L_, and inhibitor of apoptosis proteins (IAPs) XIAP and cIAP1/2 [36-38]. Together with the pro-apoptotic effects of p75, these findings show that p75^NTR^ can act as a bifunctional switch directing the cell down opposing paths of cell death or survival.

There is a wide array of proteins that have been demonstrated to interact with the intracellular domain of p75^NTR^. These include neurotrophin receptor-interacting MAGE homolog (NRAGE), p75^NTR^-associated cell death executor (NADE) and neurotrophin receptor-interacting factor (NRIF), which are all involved in mediating pro-apoptotic effects; FAP-1, receptor interacting protein 2 (RIP2) and tumor necrosis factor receptor associated factor 6 (TRAF6), which are involved in mediating pro-survival effects, while Schwann cell factor-1 (SC-1) is involved in mediating cell cycle effects and RhoA affects neuritogenesis [18]. In addition, p75 has been reported to interact with other TRAF family members, particularly TRAF2 and TRAF4, eliciting different effects on NF-κB activation and apoptosis [39].

Trk receptors are structurally unrelated to p75^NTR^ and are receptor tyrosine kinases [40]. In contrast to p75^NTR^, Trk receptors are selective in which neurotrophins they will bind, with TrkA preferring NGF, TrkB preferring BDNF and NT-4/5 and TrkC preferring NT-3 [40]. Binding of their dimeric ligands induces receptor homodimerization and trans-autophosphorylation in the intracellular domain leading to activation of three well characterized intracellular signal transduction pathways, including Ras/mitogen-activated protein kinase (MAPK) signaling, phosphatidylinositol 3-kinase (PI3K)/Akt and phospholipase Cγ (PLCγ) [28,41]. These pathways are best studied in neuronal cells, where they are involved in neuronal growth, survival and differentiation [40]. In non-neuronal cells, activation of Trk receptors is linked to proliferation, survival, migration and increased invasiveness of cancer cells (Figure 1) [42,43].

In addition, TrkA signaling has also been linked to induction of autophagy in cancer cells. For example, autophagy was identified as a novel mechanism of NGF-induced cell death via TrkA in the human glioblastoma cell line, G55 [44]. Several characteristics of autophagy were observed, including autophagic vacuoles, acidic vesicular organelles (which could be prevented by bafilomycin A1), increased processing of LC3, lack of detection of caspase activation, and vacuolation was prevented by the autophagy inhibitor 3-methyladenine [44]. The authors also found that activation of ERK and c-Jun N-terminal kinase, but not p38, were involved in autophagic vacuolation [44]. Very recently, TrkA has been shown to induce cell death in medulloblastoma Daoy cells without caspase activation and demonstrating several clear signs of autophagy [45]. However, siRNA silencing of four proteins essential to autophagy (beclin-1, Atg5, LC3 and Atg9) neither blocked NGF-induced vacuole formation or cell death [45]. Instead, the authors revealed that it is the hyperstimulation of macropinocytosis, a form of bulk fluid endocytosis [46], that is responsible for their death and that it requires TrkA-dependent activation of casein kinase 1 [45]. In both of these studies, TrkA was ectopically expressed in the cells. In fact, a series of studies by Kim and colleagues demonstrates that overexpression of TrkA in U20S osteosarcoma and SK-N-MC neuroblastoma cells induces cell death even in the absence of NGF and that, at least in U2OS, a large portion of this cell death is caused by induction of autophagy [47]. Together, these studies suggest that TrkA expression might confer a growth disadvantage to certain cancerous cells, such as glioblastoma [44], medulloblastoma [45] and osteosarcoma [47]. However, further research is required to ascertain the precise mode of death. Nevertheless, TrkA activation may potentially provide a therapeutic avenue, particularly as several current chemotherapeutics also induce autophagic cell death, e.g., [48].

## 3. NGF Signaling in Cancers

The relevance of neurotrophins to tumor biology is not well-characterized, although there are clear links: NGF was originally purified from a sarcoma, TrkA was discovered in a human colon carcinoma biopsy and p75^NTR^ was purified from a human melanoma cell line [49-51]. Altered neurotrophin signaling has since been implicated in the development and progression of a number of cancers, including neuroblastoma, medulloblastoma, melanoma, papillary thyroid carcinoma, pancreatic cancer, prostate cancer, and breast cancer (Table 1) [14]. Neurotrophin signaling in the pathogenesis of cancer has been linked to stimulation of mitogenesis, promotion of metastasis and invasiveness, and inhibition of apoptosis (Figure 1) [14,52-54]. Breast cancer and prostate cancer are notable because there is a large body of evidence demonstrating how NGF in particular is involved in disease development and progression [52,53]. In addition, neurotrophin signaling in these two cancers provides an example of how the same ligand/receptor interactions can stimulate different cellular outcomes depending on the cell type. Here we will discuss the current literature regarding the role of NGF in cell death and survival signaling in breast and prostate cancers.

### 3.1. NGF and Breast Cancer

Breast cancer is the most common malignancy in women, and is a major cause of cancer-related death in women, second in mortality only to lung cancer [80]. Advances in understanding of the disease, and development of therapeutics targeting estrogen and epidermal growth factor signaling, correlate with a notable reduction in breast cancer mortality levels during the past 10 years, with adjuvant chemotherapeutic agents such as anthracyclines, taxanes and cyclophosphamides also becoming intrinsic to cancer treatment regimes. Normal breast epithelial cells express TrkA and p75^NTR^ [55]. The function of these receptors in breast development and physiology has not been studied and in fact, ligands for these receptors are not normally expressed by adult breast cells, although it is not clear whether the receptors are activated by NGF secreted in a paracrine fashion. In contrast, breast tumors have been shown to express NGF. For example, immunohistochemical analysis has shown that NGF is expressed in up to 80% of breast cancer tumor biopsies [81]. This expression does not discriminate between breast tumor subtype and NGF mRNA levels are expressed at levels comparable to those seen in the MDA-MB-231 breast cancer cell line [81]. In another study, investigation of the expression of NGF and its receptors in breast cancer cells in effusions and solid tumors revealed that activated TrkA (*i.e.*, phosphorylated TrkA) is upregulated in effusions compared to primary breast tumors and lymph node metastasis, with downregulation of p75 in effusions [82]. Another recent immunohistochemical analysis has shown expression of NGF in fat tissue extracts from high risk patients [83]. Thus far, NGF expression has not been associated with expression of any known prognostic factor, such as estrogen receptor (ER), progesterone receptor (PR) or human epidermal growth factor receptor 2 (HER2). Anti-estrogens such as tamoxifen are used in approximately two thirds of breast cancers, while HER2 is overexpressed in 25–30% of human metastatic breast cancers [84]. Since NGF is expressed in 80% of breast cancers, this suggests that NGF signaling has a potentially broader target range than current therapies, which are directed against either estrogen signaling or HER2. Importantly, NGF can stimulate mitogenesis and pro-survival signaling in triple negative breast cancer cells (MDA-MB-231) which lack ER, PR and HER2 and are the most difficult type of breast tumor to treat [42]. Importantly, a xenograft model of breast cancer using MDA-MB-231 cells is responsive to anti-NGF treatments, including antibody and siRNA downregulation of NGF [81].

**Figure 2.**
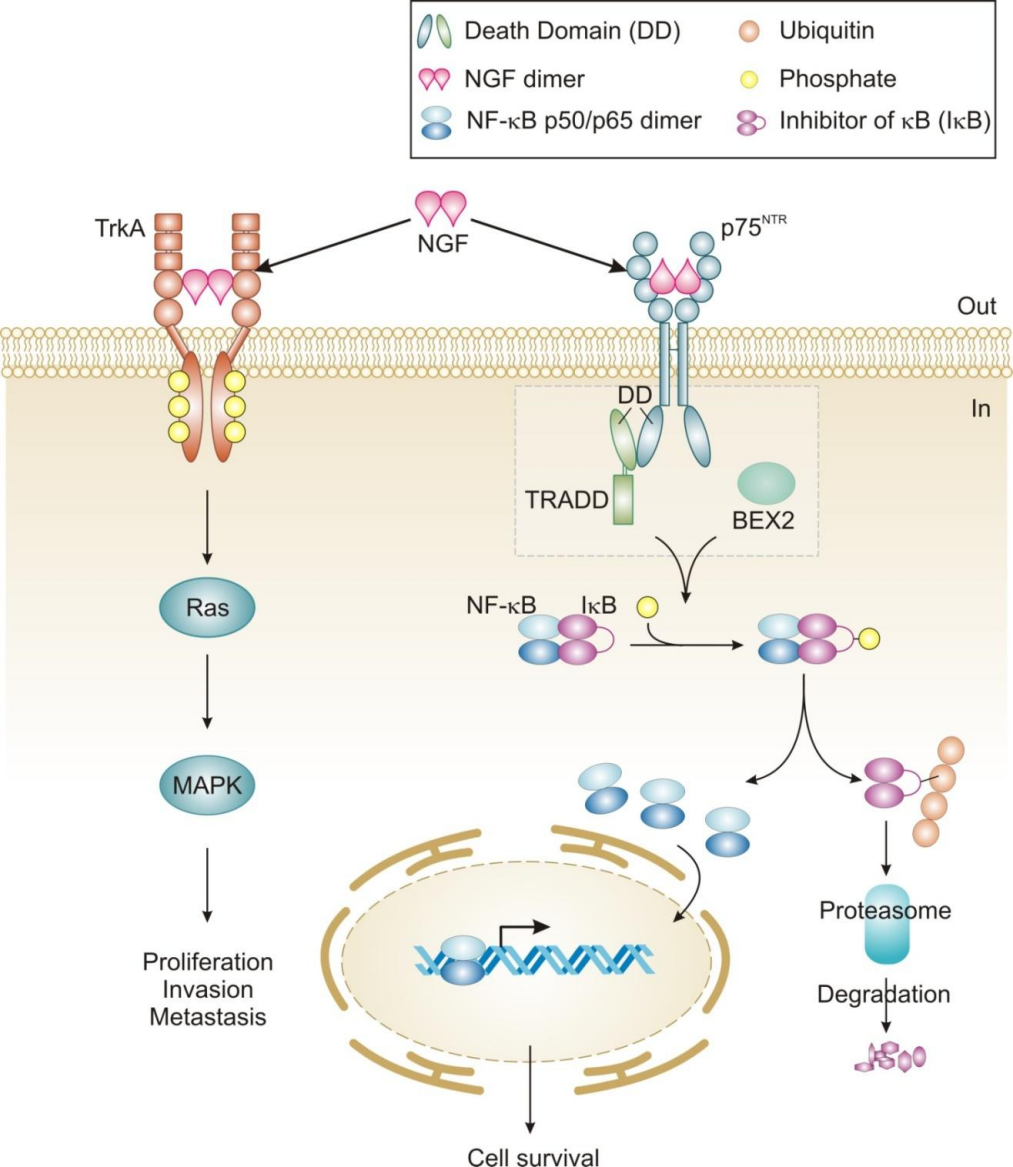
Nerve growth factor (NGF) signaling in breast cancer cells. NGF mediated autocrine signaling in breast cancer cells is carried out by two independent signaling pathways via TrkA and p75^NTR^. TrkA signals downstream to Ras/MAPK promoting proliferation of cells, as well as invasion and metastasis. p75^NTR^ signaling results in activation of the transcription factor NF-κB p50/p65 canonical pathway. The precise mechanism leading to NF-κB activation is not fully understood but is known to include TRADD and BEX2. This activation of NF-κB has been shown to be antagonized by the small peptide inhibitor SN-50. Activation of these pathways ultimately results in changes at transcriptional level. PI3K, Phosphatidylinositol-3-kinase; MAPK, Mitogen activated protein kinase; NF-κB, Nuclear factor-κB; IκB Inhibitor of κB; TRADD, Tumor necrosis factor receptor-associated death domain; BEX2, Brain expressed X-linked 2; DD, death domain; Ub, ubiquitin; P, Phosphate.

NGF signaling has been implicated in the proliferation and survival of breast cancer cells and more recently, migration [42,43,85]. The proliferative effect is via TrkA signaling and can be inhibited by tyrosine kinase inhibitors such as the indolocarbazole K252a [42]. Interestingly, tamoxifen has also been reported to inhibit NGF mediated TrkA phosphorylation in an estrogen receptor-independent manner [86]. TrkA-induced proliferation of breast cancer cells proceeds through activation of MAP kinase signaling leading to ERK phosphorylation and an NGF-dependent decrease in cell cycle duration (Figure 2) [42]. In contrast, NGF does not activate MAP kinases in normal breast epithelial cells and is not mitogenic for these cells [55]. The reason for this lack of MAP kinase activation in normal breast epithelial cells is not clear, since these cells express TrkA [55]. Recently, TrkA signaling was also shown to play a role in breast cancer metastasis and angiogenesis, which expands the role of NGF signaling in this disease [43,58].

### 3.2. NGF Pro-Survival Signaling in Breast Cancer Is Mediated by p75^NTR^

NGF exerts pro-survival effects on a wide range of breast cancer cell lines (MCF-7, MDA-MB-231, T-47D and BT-20) and can protect them from C2-ceramide induced apoptosis [42,61]. In contrast to mitogenic signaling, NGF pro-survival effects in breast cancer cells are mediated by the p75^NTR^ receptor [42]. The precise mechanism by which NGF signaling through p75^NTR^ protects breast cancer cells is largely unknown but functional studies have shown that it is mediated by activation of NF-κB [42]. In breast cancer cells the adaptors that link NGF/p75^NTR^ receptor activation to NF-κB and pro-survival signaling are largely unknown. In MCF-7 cells neurotrophin-dependent recruitment of TNF-R-associated death domain (TRADD) to p75^NTR^ has been shown to be required for activation of NF-κB and pro-survival signaling (Figure 2) [60]. Other death receptors, such as TNF-R1 and TRAIL, which recruit TRADD to their DDs, use it as a platform for the recruitment of additional adaptor proteins including receptor-interacting protein 1 (RIP1), TNF-R-associated factor 2 (TRAF2) and Fas-associated death domain (FADD) to promote either cell survival or death [87]. In fact, knockdown of TRADD causes cells to be deficient in both TNFα-induced NF-κB activation and in caspase-8-dependent apoptosis [88]. Notably, there is currently no evidence that TRADD associated with p75^NTR^ can recruit these or other adaptors in breast cancer cells or other cells. Instead, in Schwann cells, RIP2 has been shown to interact directly with the DD of p75^NTR^ and to stimulate pro-survival signaling through activation of NF-κB [89]. In addition, certain members of the TRAF family (TRAF2, 4, 6) have been reported to interact directly with the p75^NTR^ intracellular domain [39,90-92]. The role of TRAF proteins in p75^NTR^-mediated pro-survival signaling in breast cancer is not known and may be an avenue worthy of investigation.

In breast cancer cells, a novel adaptor Bex2 has recently been shown to be required for NGF/p75^NTR^-dependent NF-κB activation and prosurvival effects (Figure 2) [61,63]. BEX2 is a 15 kDa protein which is over-expressed in ER-positive breast cancer cells and is suggested to be an estrogen-regulated gene [61]. Although it has not been shown whether or not BEX2 interacts directly with p75^NTR^, it is reported to be necessary and sufficient for the anti-apoptotic function of NGF in breast cancer cells [61,62]. Furthermore, it has been shown to protect breast cancer cells from mitochondrial-mediated apoptosis, through modulation of Bcl-2 family member proteins, including up-regulation of anti-apoptotic Bcl-2 and down-regulation of pro-apoptotic members Bad, Bak and PUMA [62]. Interestingly, the homologues BEX1 and BEX3 have also been shown to interact with p75^NTR^, and in neural tissues BEX1 inhibits NF-κB, links neurotrophin signaling to the cell cycle and may sensitize cells to apoptosis [93,94]. BEX2 has also been shown to be required for progression of MCF-7 breast cancer cells through the G_1_ phase of the cell cycle via its regulation of cyclin D1 and p21 [62,63].

### 3.3. Neurotrophin Signaling in Prostate Cancer

Prostate cancer is the most commonly diagnosed solid tumor in men and the second leading cause of male cancer related deaths [80]. Its incidence significantly increases with age, regardless of variables such as diet, occupation and life style [95]. The normal adult prostate is composed of ducts with epithelial cells associated with stromal cells. The epithelial cells are of three types; secretory luminal cells which line the lumen of the duct and are surrounded by a continuous layer, formed mainly by basal epithelial cells and a scattering of neuroendocrine cells. The stroma is separated from the epithelial cells by the basal lamina, and is mainly composed of smooth muscle cells. In human prostate cancer, there is progressive disorganization of luminal and basal epithelial cells, leading to breakdown of the basal lamina and eventual metastasis of the cells to other body sites [54]. While the tumor cells are initially dependent on androgen for their survival, this dependence is progressively lost leading to development of resistance to androgen-targeting therapies. Interestingly, the secretory luminal cells are androgen-sensitive, while the basal epithelial cells express low levels of androgen receptor and the neuroendocrine cells are androgen insensitive, [54].

After the central nervous system the human prostate is the next most abundant source of NGF [96], where it plays a role in normal prostate development [97]. Stromal cells secrete NGF which binds to TrkA and p75^NTR^ present on prostate epithelial cells stimulating their growth [98-100]. It is of interest to note that BDNF, which can bind to p75^NTR^, has also been shown to be expressed by normal prostate stromal cells [101].

Progression of prostate cancer is accompanied by modifications in the expression of neurotrophins, including NGF, and neurotrophin receptors [95,102]. The most striking and important change in this regard is a reduction in p75^NTR^ expression by the epithelial cells [73]. While there is no change in the expression of NGF or TrkA, the epithelial layer exhibits a reduction in p75^NTR^ expression [73]. Immunocytochemical staining of both normal and malignant prostate epithelial tissue shows progressive loss of p75^NTR^ expression is associated with tumor development [98]. Importantly, the loss of p75^NTR^ is directly related to the grade of malignancy with early stages (prostatic intraepithelial neoplasia) still showing p75^NTR^, and poorly differentiated carcinomas having undetectable levels [103]. This is also seen in prostate cancer cell lines, where p75^NTR^ is absent from cell lines derived from advanced metastatic prostate cancer [73,74,98]. These studies also show that TrkA receptor expression is unchanged, resulting in an overall decrease in the p75^NTR^:TrkA ratio during prostate cancer progression. This is also accompanied by an increase in the *in vitro* invasive capacity of human prostatic cancer cells in xenograft models [104]. This has been reported to involve increased expression of heparanase [103]. In contrast, the mitogenic action of NGF on prostate cancer lines is mediated by TrkA [105].

There is also some evidence to suggest that during progression of prostate cancer epithelial cells acquire the ability to express neurotrophins [101]. For example, the non-metastatic LNCaP cell line does not express neurotrophins, while metastatic lines DU145 and PC-3 secrete measurable amounts of NGF [99,104] and PC-3 secretes both NGF and BDNF [99]. This switch from paracrine to autocrine control of neurotrophin activity, as well as loss of p75^NTR^, could facilitate survival and proliferation of these cells upon metastasis to other regions of the body.

This acquired ability of prostatic cancer cells to evade cell death has lead to p75^NTR^ being proposed as a tumor suppressor in prostate cancer cells [71,72,106]. In the absence of p75^NTR^, prostatic cancer cells respond to proliferative signals mediated by TrkA activation and proliferate. In fact, treatment of prostate tumor cells with pharmacological inhibitors of TrkA signaling, including K252a and CEP-701, reduces proliferation induced by NGF and leads to increased cell death [107,108]. When p75^NTR^ is artificially reintroduced into prostate cancer cells, the cells exhibit cell cycle arrest, accumulating in S phase, and undergo an increase in spontaneous apoptosis [71,105,109]. The mechanism of induction of apoptosis by NGF/p75^NTR^ has not been fully elucidated. However, reintroduction of p75^NTR^ expression into PC-3 cells is accompanied by a decrease in activation of NF-κB and c-Jun *N*-terminal kinase (JNK), suggesting these signals mediate the anti-apoptotic effect in the absence of p75^NTR^ [110]. Although pro-apoptotic signaling by p75^NTR^ in prostate cells requires the DD [71], it does not involve activation of initiator caspases-8 or -10 which are activated by other death receptors such as TNF-R, TRAIL receptors and Fas [111,112].

The loss of p75^NTR^ in prostate cancer does not appear to be due to errors or mutations in the genetic transcript coding for p75^NTR^. Rather, it has been linked to instability of the mRNA, which may be due to changes in the 3′-untranslated region (3′-UTR) of the p75^NTR^ gene [73]. The 3′-UTR of genes generally contains several regulatory sequences involved in poly-adenylation, mRNA stability and binding sites for micro-RNAs (miRNAs) which are involved in controlling mRNA stability and translation [113,114]. Thus, for example, it is possible that enhanced expression of miRNAs that target the 3′-UTR of p75^NTR^ could be responsible for downregulation of expression of the protein. Interestingly, gonadotrophin releasing hormone (GnRH) analogue therapy induces a significant increase in p75^NTR^ protein expression (with no increase in TrkA expression) [115], prompting the question as to whether GnRH analogue increases the stability of p75^NTR^ mRNA.

## 4. NGF Signaling as a Therapeutic Target in Cancer

Our knowledge of NGF signaling in various cancers suggest therapeutic opportunities. For example, in breast cancer therapies aimed at interfering with NGF/p75^NTR^ pro-survival signaling could increase effectiveness of cyto/genotoxic drugs used as adjuvant therapies in breast cancer treatment, thus lowering the dose required and reducing side effects associated with this adjuvant therapy. This is important because current combination therapies often produce unwanted side effects, e.g., doxorubicin can cause myelosuppression and cardiomyopathies [116].

NGF signaling can be targeted in a number of ways, the most common methods employ mechanisms to interfere with NGF binding to its receptors or with subsequent activation of the receptors. In prostate cancer, where the p75^NTR^ receptor is lost, there is emphasis on inhibition of TrkA proliferative signaling. In fact, strategies that are aimed at inhibiting NGF signaling are already in clinical trials for the treatment of prostate cancer [117]. In this context, pharmacological inhibitors of Trk signaling, such as derivatives of the pan-Trk inhibitor K252a, have proven interesting. One such compound, CEP-701, has been reported to block the invasive capability of prostate cancer cells *in vivo* [118]. The oral homologue, CEP-751, has been reported to induce apoptotic death of malignant cells, to decrease metastasis and to enhance host survival in *in vivo* experimental models of prostate cancer [119,120].

Small molecule inhibitors of p75^NTR^ signaling have also been produced [121]. Pep-5 is an 11 amino acid peptide that targets the intracellular domain of p75^NTR^ and inhibits it [122]. It is available as a Tat-fusion peptide which facilitates cellular entry. Also of interest are Ro-08 2750 and PD90780, small molecules that interact with NGF and prevent its binding to p75^NTR^, have proved useful in some research settings [123,124]. Thus far, these have not been fully investigated as a potential basis for therapeutic inhibition of NGF binding to p75^NTR^.

In a xenograft model of breast cancer, antibodies against NGF were successful in reducing tumor growth. This suggests that anti-NGF antibody therapies may prove useful in the treatment of breast cancer where overexpression of NGF is a factor. Furthermore, anti-NGF antibodies have been shown to reduce cell migration by up to 40% in two prostate cancer cell lines (DU-145 and PC-3), which have lost expression of p75^NTR^ and retained TrkA tyrosine kinase activity [125]. Of relevance to this point is Tanezumab, a humanized recombinant anti-NGF. Tanezumab reached Phase III clinical trials, proving highly effective at blocking pain perception in patients with chronic pain from osteoarthritis of the knee [126]. Although the trial was prematurely halted in June 2010, because some participants presented with increased damage to joints, it has been suggested that the reason for these adverse effects was excessive use of the joints as a result of lack of pain sensation [127].

Rapid advances in drug design, particularly rational design, make it likely that other approaches will be used to interfere with aberrant NGF/TrkA/p75^NTR^ signaling in cancers. For example, the development of small molecules to interfere with protein:protein interactions or of antibodies to target receptors may prove useful in treatment of these diseases. Furthermore, it is likely that new revelations regarding regulators of protein expression, such as miRNAs, or epigenetic modulators such as histone deacetylase inhibitors and DNA methyltransferase inhibitors, will reveal novel ways to target aberrant signaling due to altered expression of culprit proteins in cancers.

## Figures and Tables

**Table 1.** Dysregulation of nerve growth factor (NGF) signaling in different cancers.

**Tissue type**	**Dysregulation in NGF signaling**	**Cellular response**	**Ref.**
***Non-neuronal carcinomas***			
Breast	Gain in NGF expression via autocrine and possibly paracrine mechanisms	TrkA signaling increased leading to proliferation, mitogenesis, invasion, metastasis, angiogenesis by activating MAPK, PI3K/Akt and PLCγ	[43,55-59]
		p75^NTR^ signaling leading to activation of NF-κB and increased survival of breast cancer cells	[42,60-63]
Melanoma	NGF-mediated paracrine signaling	NGF acts as a cytostatic or differentiation factor, promoting proliferation, migration and invasion of melanoma cells.	[64-66]
	Malignant melanoma cells express p75^NTR^	Melanoma cells also express the p75^NTR^ co-receptor sortilin which with pro-NGF stimulates migration	[66]
Pancreatic	NGF expression is increased	NGF enhances proliferation invasion and tumorigenicity	[67,68]
Papillary thyroid carcinoma	Fusion of TrkA gene with various activating genes	Forms a chimeric receptor which displays constitutive activity, leading to transformation of cells	[69,70]
Prostate	Loss of p75^NTR^ protein in basal epithelial cells due to mRNA instability leading to imbalance of TrkA:p75^NTR^ ratio	p75^NTR^ proposed to be a tumor suppressor in prostate cells, so its loss facilitates survival, proliferation and metastasis of tumor cells	[71-75]
***Neuronal carcinomas***			
Neuroblastoma	Expression of different TrkA isoforms can have differing prognosis.	TrkA III isoform which is constitutively actively promotes survival via PI3K-AKT with resulting negative effects on prognosis.	[76]
		Expression of full length TrkA has positive prognosis in neuroblastoma patients.	[77]
	p75^NTR^ can induce apoptosis in neuroblastoma cell lines	NGF can induce apoptosis via p75^NTR^ in a human neuroblastoma cell line, seen with an increase in NF-κB p65 activity	[78]
Glioblastoma	TrkA	Induction by NGF of autophagic cell death	[44]
Medulloblastoma	TrkA expression has good prognosis	NGF/TrkA signaling is found to correlate with apoptotic index in primary samples. NGF induces massive apoptosis in medulloblastoma cell lines expressing TrkA	[77,79]
